# Global Biogeography of Reef Fishes: A Hierarchical Quantitative Delineation of Regions

**DOI:** 10.1371/journal.pone.0081847

**Published:** 2013-12-30

**Authors:** Michel Kulbicki, Valeriano Parravicini, David R. Bellwood, Ernesto Arias-Gonzàlez, Pascale Chabanet, Sergio R. Floeter, Alan Friedlander, Jana McPherson, Robert E. Myers, Laurent Vigliola, David Mouillot

**Affiliations:** 1 Institut de Recherche pour le développement (IRD), UR 227- Labex CORAIL, Laboratoire Arago, Banyuls/mer, France; 2 Centre de Synthèse et d'Analyse sur la Biodiversité (Fondation pour la Recherche en Biodiversité), Immeuble Henri Poincaré, Domaine du Petit Arbois, Aix-en-Provence, France; 3 Australian Research Council Centre of Excellence for Coral Reef Studies and School of Marine and Tropical Biology, James Cook University, Townsville, Australia; 4 Laboratorio de Ecología de Ecosistemas de Arrecifes Coralinos, Departamento de Recursos del Mar, Centro de Investigación y de Estudios Avanzados del Instituto Politécnico Nacional, Unidad Mérida, Cordemex, Mérida, Yucatán, Mexico; 5 Institut de Recherche pour le développement (IRD) UR 227 “CoReUs” - Labex CORAIL, Ste Clotilde, La Réunion, France; 6 Marine Macroecology and Biogeography Lab, Depto. Ecologia e Zoologia, Universidade Federal de Santa Catarina, Florianópolis, SC, Brazil; 7 Department of Biology, University of Hawaii, Honolulu, Hawaii, United States of America; 8 Centre of Conservation Research, Calgary Zoological Society, Calgary, Alberta, Canada; 9 Department of Biological Sciences, Simon Fraser University, Burnaby, British Columbia, Canada; 10 Seaclicks/Coral Graphics, Wellington, Florida, United States of America; 11 Institut de Recherche pour le développement (IRD) UR 227 “CoReUs” - Labex CORAIL, Noumea, New Caledonia; 12 Ecologie des Systèmes Marins Côtiers, ECOSYM UMR 5119, Université Montpellier 2, Montpellier, France; Aristotle University of Thessaloniki, Greece

## Abstract

Delineating regions is an important first step in understanding the evolution and biogeography of faunas. However, quantitative approaches are often limited at a global scale, particularly in the marine realm. Reef fishes are the most diversified group of marine fishes, and compared to most other phyla, their taxonomy and geographical distributions are relatively well known. Based on 169 checklists spread across all tropical oceans, the present work aims to quantitatively delineate biogeographical entities for reef fishes at a global scale. Four different classifications were used to account for uncertainty related to species identification and the quality of checklists. The four classifications delivered converging results, with biogeographical entities that can be hierarchically delineated into realms, regions and provinces. All classifications indicated that the Indo-Pacific has a weak internal structure, with a high similarity from east to west. In contrast, the Atlantic and the Eastern Tropical Pacific were more strongly structured, which may be related to the higher levels of endemism in these two realms. The “Coral Triangle”, an area of the Indo-Pacific which contains the highest species diversity for reef fishes, was not clearly delineated by its species composition. Our results show a global concordance with recent works based upon endemism, environmental factors, expert knowledge, or their combination. Our quantitative delineation of biogeographical entities, however, tests the robustness of the results and yields easily replicated patterns. The similarity between our results and those from other phyla, such as corals, suggests that our approach may be of broad utility in describing and understanding global marine biodiversity patterns.

## Introduction

Delineating regions is a critical step in biogeography if we wish to understand the historical and evolutionary forces shaping biodiversity patterns [1]. From an applied point of view, this delineation is also very important in the setting of conservation priorities based on the composition of species assemblages [2]. Defining marine biogeographical regions on a global or large regional scale has been proposed by a number of authors. However, biogeographical delineation based on quantitative approaches remains a challenging task owing to the difficulties in obtaining and analyzing spatially comprehensive data. This has resulted in many qualitative approaches, leading to multiple delineations differing in the number, size and boundaries of the biogeographical regions.

Ekman [3] was the first to define biogeographical regions of the marine realm, based upon zoogeographical characteristics, environmental barriers, and levels of endemism. Briggs [4], working with similar concepts, set a minimal level of 10% endemism for defining regional faunas. Briggs and Bowen [5] revised this earlier analysis by considering recent advances in our knowledge of the geographical distribution of species and their phylogenetic relationships. Other studies have identified major barriers that set boundaries to biogeographical regions. For example, Bellwood and Wainwright [6] proposed major hard barriers (Red Sea land bridge, Isthmus of Panama) and a number of soft barriers (e.g. Eastern Pacific Barrier, Amazon-Orinoco, Cape Province, South India-Maldives, Indian-Pacific Oceans, California, Salvador). Even though these barriers implicitly define regions, their biogeographical significance was not formally tested (see [7] for methods).

Other works, explicitly proposing a biogeographical classification of regions were based on expert knowledge [8], or used environmental variables [9], [10]. Following these approaches, Spalding et al. [11] defined biogeographical realms, provinces, and eco-regions based on a compilation of existing work on delineating biogeographical entities, combined with expert knowledge, for several phyla.

Reef fishes represent one of the best candidates to conduct a quantitative assessment of biogeographical regions worldwide owing to their high diversity (with nearly 6,500 species), their well-known taxonomy [12], [13], [14], and the well-documented geographical distributions of a very large number of species. Thresher [15] provided the first attempt to statistically analyze relationships between marine areas based on relative species richness of 46 reef fish families; however, he did not define biogeographical regions. Likewise, Bellwood and Wainwright [6] delineated areas based upon species richness but also did not specifically define biogeographical regions. More recently, Floeter et al. [14] used reef fish checklists across the Atlantic to define biogeographical regions using parsimony analysis to match the regional boundaries with known geographical barriers. Similar approaches have also been used by Robertson and Cramer [16] to define biogeographical regions in the Eastern Tropical Pacific (ETP) and by Kulbicki [17] in the South Pacific. These quantitative studies, however, have either been spatially restricted, did not take into account species identity, or did not define distinct biogeographical regions. Obtaining consistent, quantitative, biodiversity patterns remains crucial in testing hypotheses about the spatial organization or large-scale functioning of reef fish assemblages. For example, the “Coral Triangle” or Indo-Australian Archipelago (IAA) is the subject of much debate on the role of speciation and dispersal processes in shaping this biodiversity hotspot e.g. [6], [18]–[25]. There is an implicit suggestion, in many studies, that the peak in reef fish diversity found within the IAA defines it as a biogeographical region often termed the ‘Coral Triangle’. However, a clear delineation of this region, based on species composition, remains elusive and needs to be quantitatively assessed. This discussion extends to the broader topic of the biogeography of reef fishes and the large-scale processes underpinning the geographical distribution patterns of species. In particular, works on connectivity [26], [27], [28], dispersal [29], [30], mid-domain [19], latitude gradients [31], [32], hot-spots [33], [12], large-scale distributions [34], evolutionary origins and dispersal over evolutionary timescales [35], [36], conservation planning [37], [38], energy input [39], [40] and biodiversity partitioning [41] could all benefit from the identification of large scale species pools corresponding to biogeographical regions that are delineated by quantitative approaches with known accuracy and precision.

There is currently no universally accepted terminology for a biogeographical hierarchy, with the most recurrent terms being realm, region, province, and eco-region, as initiated by Kaufman [42] to define paleo-biogeographical units. Terminological subjectivity is further confounded by the fuzziness between boundaries based upon endemism or species composition and those based upon environmental factors. A biogeographical terminology that reflects the delineations that are generated by quantitative analyses based upon taxonomy is therefore lacking.

Furthermore there is no uniform way to define biogeographical regions based on species composition since this delineation may depend on the question being asked. For instance, the method employed may change if one is interested in endemism, in assemblage similarity or species richness. There are, however, a number of common properties that are desirable in the methods used. For example, the quantitative analyses of species lists should not be overly sensitive to small changes in species composition and it should give some indication of the quality, or robustness, of the classification. These seemingly simple objectives have seldom been met in the classifications available to date. One reason for this lack of consistency was the lack of easily accessible statistical methods to meet these needs. With the advent of bootstrapping algorithms for the assessment of uncertainty in hierarchical classifications (e.g. [43], [44]), it is now possible to test the robustness of a classification and to compare several classifications.

This study, therefore, takes advantage of the largest dataset on reef fishes to date, with over 6,300 species distributed amongst 169 checklists, combined with recent advances in data analyses, which provide a robust, hierarchical classification of biogeographical entities. The goal of this study is to quantitatively define the biogeographical hierarchy for reef fish faunas around the world, showing the levels of linkage between regions based on their species composition. Our quantitative approach provides a foundation for developing hypotheses that seek to resolve the major questions concerning the evolutionary history and processes underpinning diversification and distribution patterns in this important group of chordates.

## Materials and Methods

To delineate biogeographical patterns in reef fishes, we assessed how different areas relate to each other, hierarchically, in terms of similarity in species composition. To do so we needed a classification that is relatively insensitive to small changes in species composition while providing a measure of the quality or robustness of our analysis. This classification can then be compared with pre-existing global and regional schemes. Our analysis required four methodological steps: (*i*) building a global database of reef fishes for the tropical regions; (*ii*) delineating biogeographical units using cluster analysis; (*iii*) assessing the robustness of area dendrograms; and (*iv*) constructing a hierarchical classification of biogeographical entities.

### Building a global database of reef fishes in the tropical regions

To conduct a quantitative assessment of biogeographical regions, it is necessary to use taxa with sufficient diversity within all regions, good taxonomical resolution (as too many uncertainties may create false robustness), and good data on geographical distributions. Reef fishes met all three criteria.

We limited our research to tropical reefs, including coral or rocky reefs, and in areas with a minimum monthly sea surface temperature (SST) of 17°C. Rocky reefs were considered in addition to coral reefs as previous studies have indicated that many coral reef fishes can inhabit both reef types [45]. The limit of 17°C was set because we decided to include locations that are not truly tropical as defined by SST>20°C [5], but where species with tropical affinities are present. The inclusion of those areas between 17°C and 20°C broadens the range of variation in species composition and assemblage dissimilarity, thereby increasing the breadth of our classification.

Within the area selected for data collection, we obtained information on species composition at 169 locations worldwide [46]. The information was obtained by examining nearly 500 references and extracting information from published works, regional checklists, monographs on specific families or genera, and reports. Elasmobranches were not considered because many have very different biological traits and evolutionary histories when compared to most reef fishes. Most elasmobranchs are live-bearers compared to less than 0.5% of reef teleosts. Their dispersal capacity is therefore linked to their adult behaviour, with most sharks having very wide distributions, whereas rays tend to be more sedentary which may increase vicariance and allopatric speciation e.g. [47], [48]. It should be noted that the global phylogeny and phylogeography of fishes [49], and reef fishes in particular, are far less advanced than in other groups of vertebrates (birds, mammals, amphibians, reptiles). For instance, despite the good level of knowledge on reef fish distributions there are still many gaps, as indicated by the high rate of species description [50], [51] and the frequent discovery of cryptic or sister species, even in well-known families [52]. The present work will investigate the impacts of these two sources of uncertainty by accounting for the reliability of knowledge on the geographical distribution of species, and the geographic coverage and sampling effort of checklists.

The current knowledge of the geographical range of a number of reef fish species may be unreliable due to species' behavioural traits (e.g. nocturnal, cryptic) or unresolved taxonomy. To test whether this potential information gap had any influence on the definition of our biogeographical delineations, species were defined as either ‘reliable’ or ‘debatable’. Clusters were then built by either using “all species” (both reliable and debatable) or “reliable” species only (see Table S1 for list of reliable families and genera).

The scope of checklists may also be important. As sampling effort, represented by the number of expeditions, or taxonomists, or number of specimens available in museums, is not homogeneous over all checklists, we may group checklists in order to lower this heterogeneity in sampling intensity. One way to achieve this grouping is to combine all species within previously defined regions. For consistency across the entire study area, we chose to use the limits of the eco-regions defined by Spalding et al. [11] as we had data for all the 111 eco-regions they defined in the tropical band. We therefore built classifications based on either individual “checklists” or on the combined “eco-regional lists”.

By distinguishing “reliable species” versus “all species” and “checklists” versus “eco-regions”, four different classifications were obtained. The degree of agreement between classifications based on “all species” and the classification based solely on “reliable species” was assessed using the Variation of Information criterion (hereafter ‘VI’) proposed by Meila [53], which measures the amount of information lost and gained in changing clustering classifications and corresponds to zero when two classifications are identical. VI was calculated by recursively cutting the dendrograms of each classification into 1 to 20 groups. The final values of VI were compared to those obtained by 100 random permutations of group memberships. After proving that all classifications converge to similar results, we chose the classification based on all species and checklists for a final delineation since this represents the most complete and independent set of data.

### Delineation of biogeographical units using cluster analysis

We delineated biogeographical units using cluster analysis and largely followed the methodological framework proposed by Kreft and Jetz [54]. Cluster analysis produces a quantitative, hierarchical classification of the dissimilarity among species assemblages, but is sensitive to the dissimilarity measure and the classification algorithm chosen.

Amongst the myriad of dissimilarity indices available (reviewed in [55]) we selected *βsim* [56] for our analysis because, unlike commonly employed dissimilarity measures (*e.g.* Jaccard, Sørensen), *βsim* is not affected by variations in species richness [55] and can be considered a pure ‘turnover’ index without the nestedness component [57]. Since species lists at our locations were compiled from areas of different sizes, dissimilarity indices affected by richness were considered inadequate as any potential effect of sampling effort should be avoided in biogeographical studies [54]. In addition, compared to other “richness-free” indices (see [55] for a review), the properties of *βsim* are well known and it has been successfully employed in a number of biogeographical analyses [54], [58], [59].

Given that our goal was to group locations into biogeographical units, we then selected the Ward agglomerative clustering method (i.e. minimum variance) because it reduces the number of singletons (i.e. clusters composed of only one location) by penalizing large groups to produce a final dendrogram with a homogenous distribution of locations among groups. The accuracy of the Ward algorithm, i.e. its ability to conserve the initial dissimilarity values between pairs of locations (low distortion), was however evaluated using the cophenetic correlation coefficient, i.e. the Pearson product moment between the cophenetic distance calculated on cluster branches and *βsim* [54]. The cophenetic correlation coefficient provides an indication of the amount of information contained in the initial dissimilarity matrix that is transferred to the cluster dendrogram. Following [60], we considered a dendrogram as valid when the cophenetic correlation coefficient was 0.8 or greater.

### Robustness of the clustering dendrograms

The quantitative delineation of biogeographical regions has many potential sources of uncertainty, which are, in particular, related to the nature of thedata. Being based on checklists of reef-associated fishes at a worldwide scale, our analysis is intrinsically influenced by the heterogeneous quality, accuracy and sampling efforts characterizing the various checklists, all of which introduce uncertainty. The robustness of the dendrograms was tested using a multi-scale multi-step bootstrap resampling technique. This method was initially devised for phylogenetic trees [43] and later extended to any clustering method [44]. We calculated robustness estimates ranging from 1% (low) to 100% (high) at each node of the dendrogram, thereby allowing us to modify our level of confidence in a cluster given its relative level of robustness. The robustness value at a given node indicates how often (in %) a partitioning below that node was observed for dendrograms obtained in bootstrap analyses. We conducted the analysis using 10,000 bootstrap replicates of dendrograms.

### Constructing a hierarchical classification of biogeographical entities

As previously mentioned, there is currently no universally accepted terminology for hierarchical biogeographical units, but the most recurrent terms are realm, region, province, and eco-region, as initiated by Kaufman [42]. We defined biogeographical realms (1^st^ level of the dendrogram), regions (2^nd^ level) and provinces (3^rd^ level) along a decreasing gradient of dissimilarity in species composition. Low and uneven robustness of nodes below the third level discouraged us from attempting a delineation of eco-regions.

## Results

**Figure 1:**
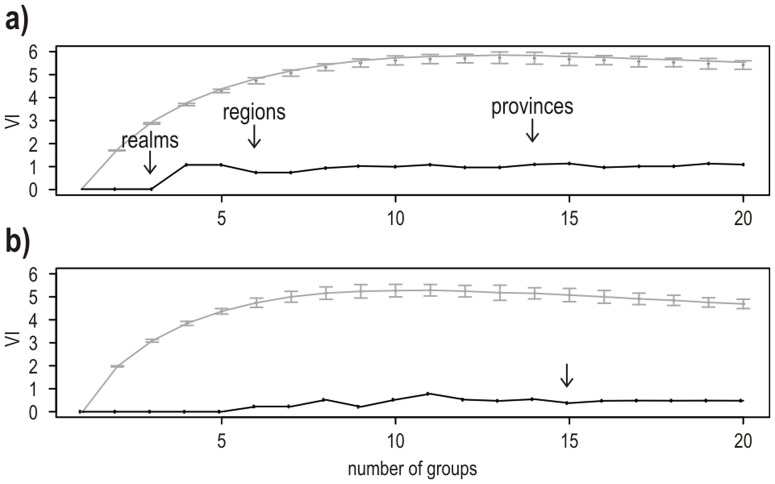
Uncertainty of cluster classifications depending on the species used (i.e. all species or reliable species). a) Values of VI (Variation of Information criterion) for clusters based upon fish checklists; b) values of VI for clusters based upon eco-regions. The arrows indicate the partitioning levels corresponding to realms, regions and provinces on figure 1a. The arrow on Figure 1b corresponds to the limit for provinces (the limits are the same as figure 1a for realms and regions). The grey lines correspond to the values of VI obtained by 100 random permutations of group membership.

**Figure 2:**
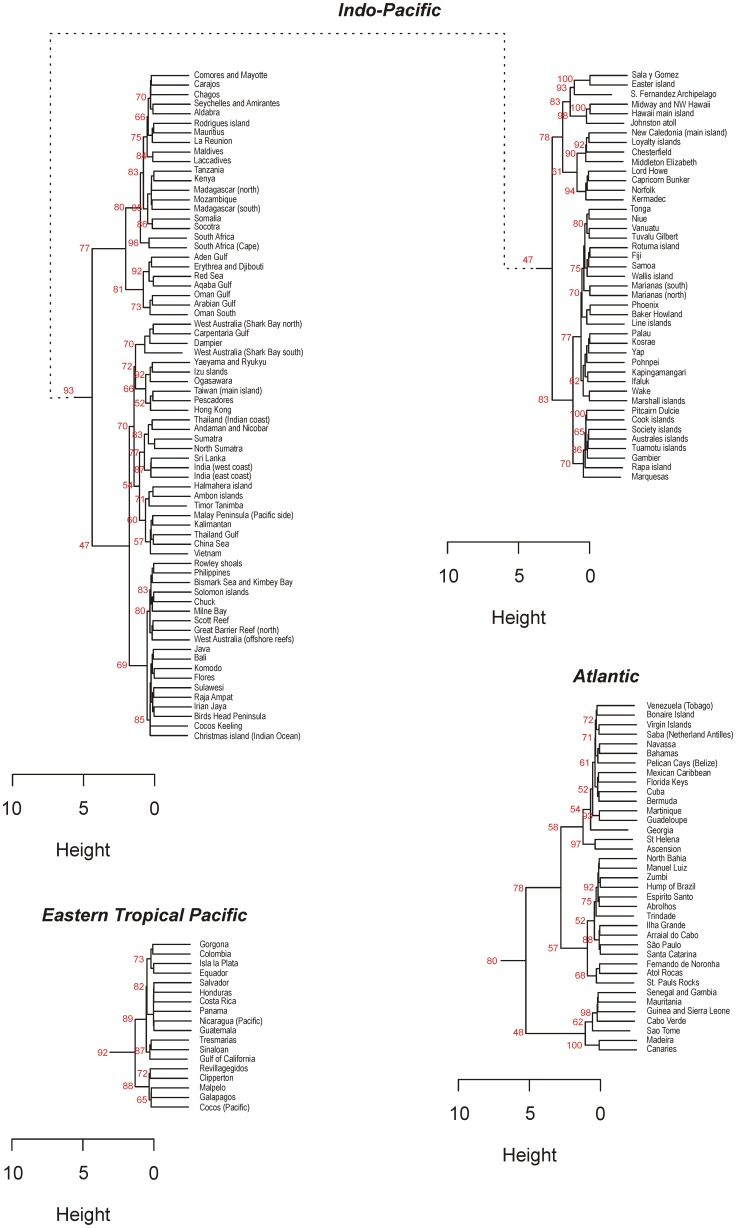
Hierarchical classifications based on species dissimilarity using checklists and all species (reliable and debatable). For clarity the three realms were separated. The values at the base of the branches indicate the % bootstrap support (i.e. the proportion of classifications obtained with bootstraps (n = 10 000) which yielded the same results).

The 169 checklists contained a total of 6,316 reef fish species out of which 2,769 were classified as “reliable”. Using “all species” or “reliable” species yielded similar results (Figure 1) as the Meila's VI was less than 1.5 when comparing clusters based on checklists, whereas random groups yielded values around 6 (Figure 1a). The VI was even lower (less than 1) when comparing clusters based on eco-regions (Figure 1b). This indicates that our four area dendrograms and subsequent classifications provide converging results. When reporting the number of species and endemics per biogeographical entity, we focused on the checklists×all species classification, as it utilized the most comprehensive dataset (Figure 2).

**Figure 3:**
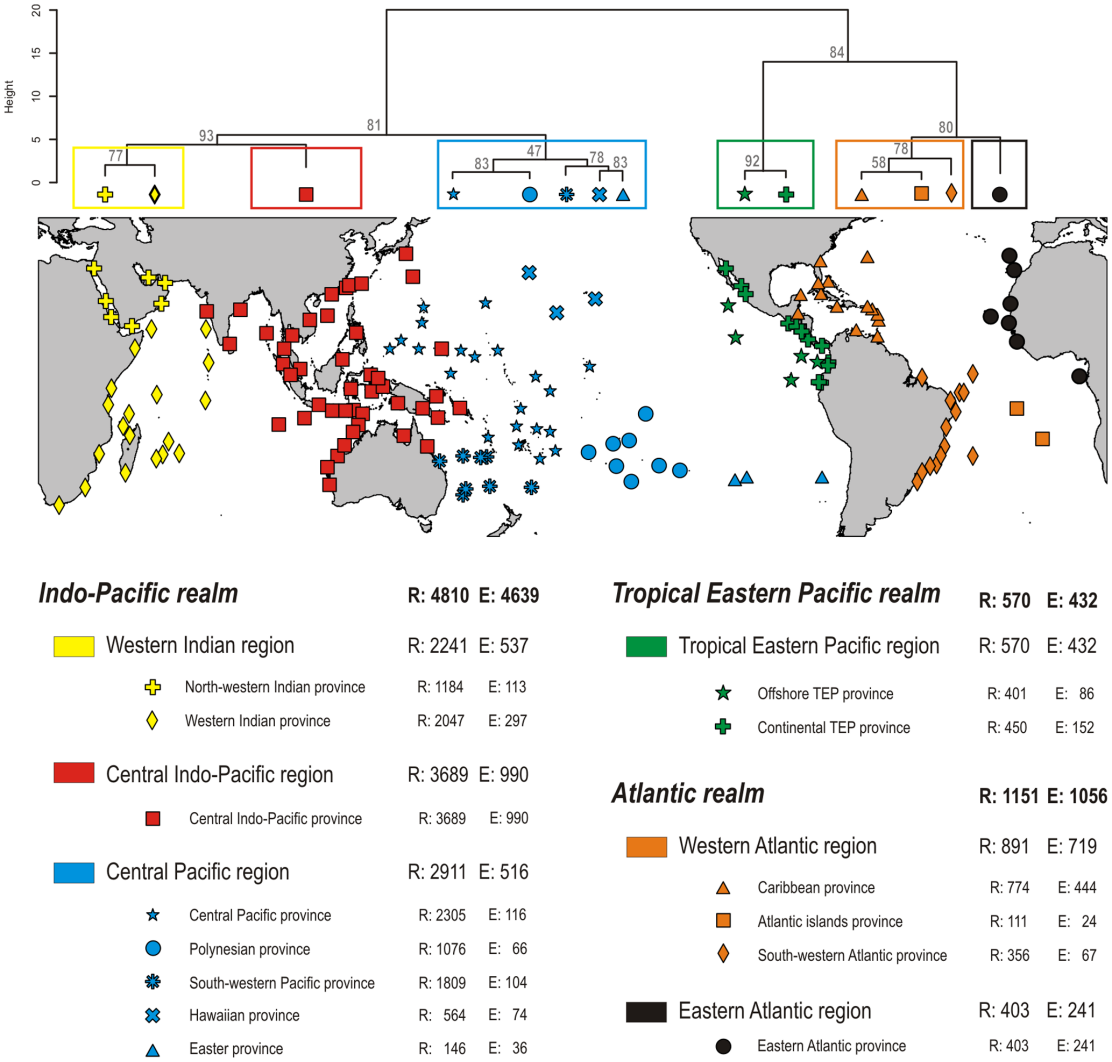
Map of the realms, regions and provinces defined by a clustering of reef fish checklists based on all species (“checklist”×“all species” data set). Each point represents one of the 169 checklists. R: number of species; E: number of species exclusive to the area considered (endemics).

All four classifications identified three identical realms: Atlantic, Eastern Tropical Pacific (ETP) and Indo-Pacific (Figure 3). The species richness within each of these realms was markedly different, with the Indo-Pacific totaling 4,810 species, the Atlantic 1,151, and ETP 570. The number of species common to all three realms was extremely low, with only 37 species found in all three realms, 84 species common to the Atlantic and Indo-Pacific, 131 species common to ETP and Indo-Pacific, and 46 species common to the ETP and Atlantic.

**Figure 4:**
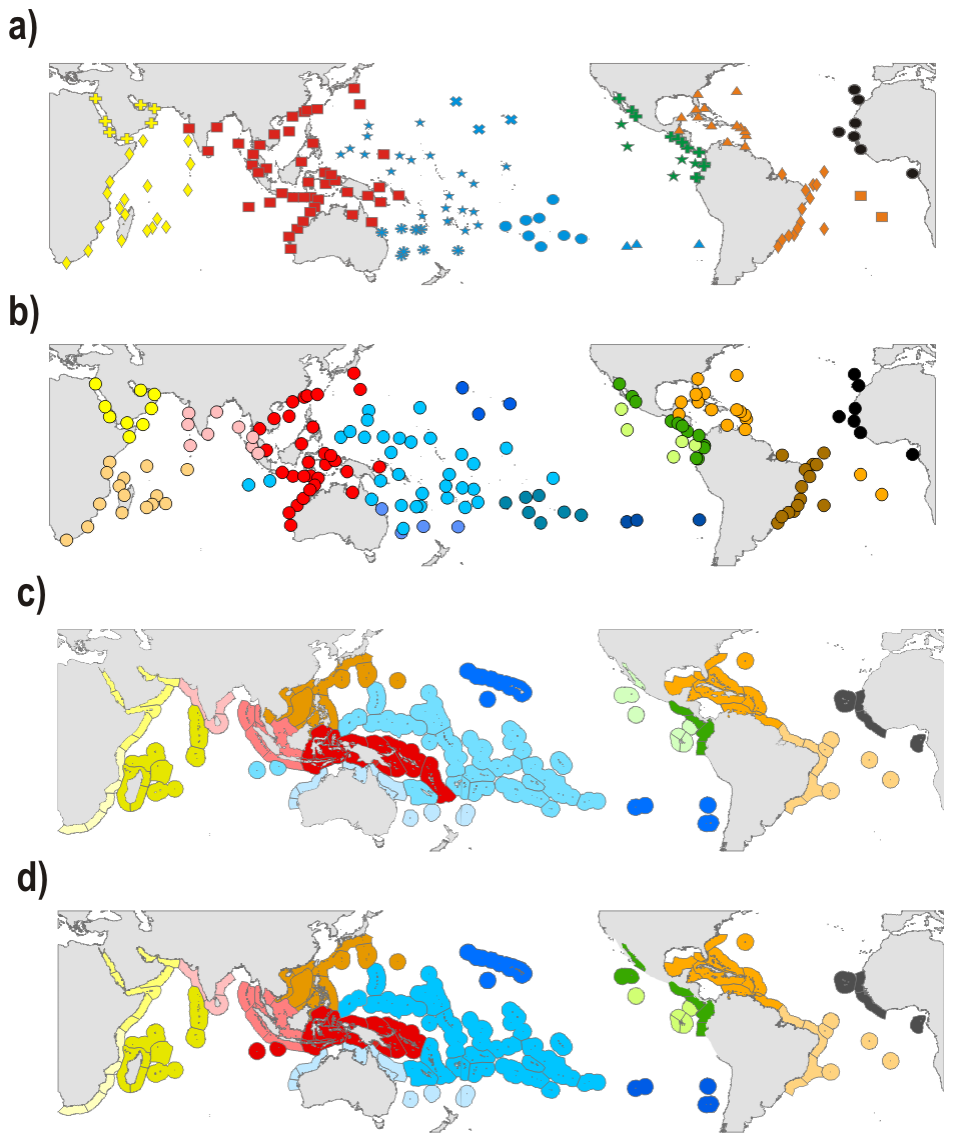
Hierarchical classification a) based on all the species and employing checklists as base units (as on Figure 3); b) based on the reliable species and employing checklists as base units; c) based on all the species and employing eco-regions as base units; d) based on the reliable species and employing eco-regions as base units.

Within each realm, the regions exhibited only minor variation among the four classifications (Figures 4 a, b, c, d). The Atlantic realm was divided into two distinct regions: the Eastern Atlantic (403 species; 59% endemism) and the Western Atlantic (891 species; 80% endemism). Although geographically separated by the mid-Atlantic barrier these two regions had a relatively high proportion of species in common (131 species; 11%). The delineation of Atlantic provinces was also largely consistent among the four classifications, the only difference being the Ascension, St. Helena and Trindade islands (South Atlantic) which either were grouped with the Caribbean (Figures 3, 4b) or with the Brazilian provinces (Figures 4 c, d) depending on the analysis. The Caribbean province was the most species rich (774 species) and had the highest percentage of endemics at the province level (57%). The South-Western Atlantic province (Brazil) had 356 species with 18% endemism and was separated from the Caribbean Province near the location of the Amazon-Orinoco fresh-water plume. The south Atlantic islands (Ascension and St. Helena) were very small and remote islands with only 111 species of which 21% are endemics.

According to all four classifications the ETP realm comprised a single region with extremely high endemism (76%). There were two provinces within the ETP with slightly different limits depending on the classification. The Continental ETP and the Offshore ETP had similar number of species (450 and 401, respectively) despite a much larger habitat area associated with the continent. The level of provincial endemism was higher in the Continental ETP (34%) than in the Offshore ETP (21%), with the latter sharing many species in common with the Central Pacific (124 species). The classifications based on eco-regions (Figures 4 c, d) gathered southern California and the offshore Mexican islands either with the Continental ETP province or with the Offshore ETP province, but all four classifications clustered Clipperton, Galapagos, Malpelo and Coco islands together within the Offshore ETP province.

Our four classifications (Figure 4) divided the Indo-Pacific realm into three regions: the Western Indian region (2,241 species), the Central Indo-Pacific region (3,689 species), and the Central Pacific region (2,911 species). The position of Australia, the Cocos-Keeling and Christmas islands was, however, unstable. With 24%, 27% and 18% endemism, respectively, the three Indo-Pacific regions had much lower level of endemism than regions in the Atlantic (59–80%) or ETP (76%). The division of regions into provinces varied depending on the classification. A general agreement was found for the Indian Ocean which was split into two provinces: a North-Western Indian province (9.5% endemism) that comprised the Red Sea and Arabian Peninsula, and the Western Indian Ocean province (14.5% endemism) that grouped the Seychelles, the Mascarene Plateau, Madagascar, and the west coast of Africa from Somalia to South Africa. Somalia, Kenya and Tanzania were grouped with the Red Sea according to the “eco-region” classifications, whereas they grouped with the Seychelles, Mascarene, SE Africa and Madagascar according to the “checklists” classifications. The Maldives, Laccadive and Chagos archipelagos, located in the central Indian Ocean, were also included in the Western Indian Ocean province, except in the checklist×reliable classification.

The Central Pacific region was divided into either five provinces of unequal areas according to the checklist classification or three provinces according to the eco-region classification. Unlike eco-region classifications, checklist classifications separated the Hawaiian Archipelago from the group composed by Easter Island, Sala y Gomez and Desventuradas islands. Checklist classifications also generated a “South Western Pacific Ocean” province grouping of Lord Howe, Norfolk and Elisabeth-Middleton reefs, whereas eco-region classifications generated an “Australian region” extending from western Australia all the way to the Kermadecs. The Central Pacific province, identified by all four classifications, was the largest in area and grouped the highest number of species (2305 species). Its level of endemism was low (5%), but comparable to the South-Western province (5.7%) and Polynesia (6.1%); both were also rich in species(1,809 and 1,076 fishes, respectively). Conversely, the Hawaiian and Easter Island provinces were small, had relatively few species (736 and 146 species, respectively), but comparatively higher levels of endemism (20.7 and 25%, respectively).

The major differences between checklist and eco-region classifications were found for the Central Indo-Pacific region which is characterized by low within-region dissimilarity. Using checklists, this region was either a single province (all species classification, Figure 4a) or comprised two provinces (reliable species classification, Figure 4b), the more western province covering India all the way to Sumatra and the other province integrating Western Australia, the IAA (Coral Triangle) and the Taiwan-Japan area. Classifications based on eco-regions produced 3–4 provinces, a western province, from the Java Sea to West India, a central province reaching from Vietnam to Japan, and one or two provinces grouping (all species, Figure 4c) or separating (reliable species, Figure 4d) the IAA and Melanesia.

Bootstrap values were higher for eco-region classifications than for classifications based on checklists (see Fig. S1 to S4 in File S1), the lowest values being observed for the checklist×all species classification. The levels of these values were correlated to the number of initial objects (checklists or eco-regions) and the number of species (classifications based on “all species” generating lower bootstrap values because more species are involved).

## Discussion

This study is a step forward from previous works on the biogeographical delineation of marine regions since it is based on a statistical analysis of the dissimilarity in species composition integrating multiple sources of uncertainty. The analyses quantified the robustness of biogeographical delineations by: (i) taking into account the quality of the data, both spatially (checklist vs. eco-region based classifications) and taxonomically (all species vs. only those with reliable, known distributions); (ii) comparing four alternative classifications; and (iii) quantifying the uncertainty of clustering results via internal bootstrapping.

The most remarkable result is the extent of concordance in the four classifications at the realm and regional levels, showing that these biogeographical entities are robust to uncertainty for reef fishes. The partitioning of regions into provinces is not as robust with several differences amongst our classifications, mainly in the Central Indo-Pacific region, which is characterized by low within-group dissimilarity. This low dissimilarity is indicated by the lower bootstrap values obtained at many nodes at the province level, especially in the Indo-Pacific. In most instances, despite these low values, the limits of these provinces matched with known “soft barriers” such as the limit of the Pacific tectonic plate (limit between Polynesia and the central Pacific provinces [61]), and the limits of the Hawaiian or the Easter Island groups, which are mainly separated by large expenses of open oceanic waters. Unless the bootstrap values are 100, the limits defined by the clusters should be regarded as “fuzzy”, the amount of fuzziness being inversely proportional to the bootstrap value. There is no specific decision rule regarding bootstrap values, however, values above 80 are considered to be useful in constructing classifications. Despite the fact that bootstrap values are obtained in a similar way to phylogenetic trees [43], our dendrograms do not directly infer evolutionary or historical associations but solely dissimilarity in species composition, although they may reflect evolutionary processes [36], [62].

Despite major methodological differences, our results do support some previous works. Kulbicki et al. [63] provide a global classification of Chaetodontidae (butterfly fish) based on a very different algorithm (Raup and Crick's distance [64]) which show many similarities with our study. In particular the Atlantic and ETP had a similar structure and the Indo-Pacific was characterized by low bootstrap values, although, as in the present study, Hawaii and Easter Island do form distinct groups. In the Atlantic, Floeter et al. [14] performed a similar analysis. They likewise separated the East from the West Atlantic and the Brazilian province from the Caribbean. The major difference is in Ascension and St. Helena which belonged to the East Atlantic in their classification, whereas these islands are associated with the West Atlantic in ours. Briggs and Bowen [5] indicate that these two islands do not have a clear and strong link to either the East or West Atlantic as they both have high levels of endemism and share species with both sides of the Atlantic.

Numerous classifications have been proposed for the ETP [16] with little agreement, except that offshore islands are usually separated from the mainland, with the Galapagos standing apart [3], [78], [4], [65]. Robertson and Cramer [16] provide several classifications based on different types of fish (all shore fish species, reef fishes, soft-bottom fishes, pelagic fishes). Their classification based on reef fishes indicates that all offshore islands are in one group, similar to three out of four of our classifications (Figures 4). Robertson and Cramer [16] divided the inshore area into a central zone spanning from Ecuador to the Baja California Gulf, and two border zones, one in the north (Baja California Gulf and Baja California) and one in the south (Peru). Our classifications did not separate the inshore ETP into several provinces, except in the eco-region×all species classification which associated the Baja California Gulf and Baja California with the offshore islands. Briggs and Bowen [5] considered these offshore islands, with the exception of Galapagos, as outposts of the “Panamanian province” because of their low endemism. As our classifications take into account not only endemism but also species in common with the Central Pacific, it is logical that the ETP offshore islands group together as many of these Central Pacific species do not reach the mainland, suggesting that a range of processes are involved in shaping entire reef fish assemblages.

The partitioning of regions into provinces was least consistent in the Indo-Pacific realm. This is due to the high degree of similarity in assemblages over large geographical scales in the Indo-Pacific. In addition, endemism is low (>7% only for the Marquesas, Red Sea, Hawaii, and Easter Island-Sala y Gomez checklists) which means that the dissimilarity level will be low and delineations of the clusters not as robust as in other realms (Fig. S1–S4 in File S1). Christmas Island and Cocos (south west of Indonesia) are typical examples of sites which show an unstable classification. In this case this is due to the joint presence of sister species from the Indian and the Pacific Oceans. For instance *Chaetodon lunulatus* (Pacific) and *C. trifasciatus* (Indian O.) are both present on these islands [66]. The Solomon Islands are another example of an unstable classification, as they are grouped with the Central Indo-Pacific when using “total species” but group with the Central Pacific if one uses only “reliable species”. This is mainly due to small sedentary species belonging to the Gobiidae and to a lesser extent Trypterygiidae, Anthiinae, Apogonidae and Blenniidae. These families could assist in making a better delineation of the regions, but they are presently still under-sampled across the globe and care is needed when using them in biogeographical work.

Briggs and Bowen [67] suggested that the area from the Gulf of Oman to French Polynesia belongs to the same biogeographical entity. Our work clearly indicates that there are biogeographical subsets within this space, but no clear barriers. The spatial organization of reef fish assemblages in this realm is structurally weak, as noted by Briggs and Bowen [67] and Kulbicki et al. [63]. Spalding et al. [11] divided this area into several biogeographical entities, e.g. the Western Indo-Pacific, the Central Indo-Pacific, the Eastern Indo-Pacific. They also defined “realms” which border what we defined as the Indo-Pacific realm (e.g. Australasia and Northern Pacific). These divisions and the subsequent partitioning into “provinces” [11] seldom match the delineations identified by our four classifications. However, this may reflect their use of non-fish taxa and environmental information. There are also some differences between our results and those of Santini and Winterbottom [20], who based their delineation on endemism. In particular, they divided the Western Indian Ocean into eight entities, whereas we have only three and they found the Australian area split into three entities whereas we have only one. Since they used a cladistic approach to cluster regions, their results are not directly comparable to ours. However, in the Indian Ocean their regions clustered similarly to our study (Red Sea with Arabian basin; the regions of East Africa come together with the Mascarene and the Chagos-Maldives; Andaman and East Indian region come together). Similarly, in the Pacific Ocean, Hawaii, the Coral Sea-Polynesia area and Micronesia were associated by Santini and Winterbottom [20] in a manner comparable to our classifications, even if the limits of their regions are different from ours. In the Indian Ocean, the study of Obura [68] based on corals showed a Red Sea-Arabian region which matches our classification. His study indicated a western Indian Ocean region which matches with the north and south of our checklist×reliable species classification (Figure 4 b). This raises the problem of the Chagos-Maldives-Laccadive area which, depending on the classification, belongs to either the western Indian Ocean or the Indian sub-continent [57]. This is in accordance with the opinion of Briggs and Bowen [5] who indicated that the position of this zone is open to debate. Santini and Winterbottom [20] suggest that this area is a link between the Mascarene and the Indian sub-continent-Andaman area. The western Indian Ocean and the Chagos-Maldives-Laccadive are on two separate tectonic plates. However, Springer [61] showed no clear evidence that these two plates have distinct faunas, even if for some species it seems to represent a biogeographical limit. Mouillot et al. [57] show that the associations in this area are particularly sensitive to the type of dissimilarity index used, with the Maldives showing stronger links to the IAA or Madagascar depending on whether analyses use total dissimilarity or just the turnover component without accounting for nestedness.

In the Pacific, our results match those from Kulbicki [17] even though he employed a similarity algorithm that included nestedness (where delineations can be confounded by changes in species richness). Interestingly, his study and ours show a convergence between the north-east (Hawaii) and south-east (Easter Island, Rapa) Central Pacific which can be related to the numerous species showing an anti-tropical distribution in this part of the Pacific [69]. Another similarity with the work of Kulbicki [17] is that the Marquesas are grouped with Polynesia and do not form a separate entity, despite their relatively high endemism (7.5%; this value is lower than that of Randall and Earl [70] who found 11.6% endemism, but based on 415 species, whereas our study has 547 species). An intriguing pattern in two of our classifications is the clustering of all of Australia's eco-regions into a single group (Figures 4 c, d) which belongs to the Central Pacific region. There are a number of species endemic to both sides of Australia, as well as species in common through connectivity along the southern coast of Australia (essentially temperate species), but most tropical species that share the two sides of Australia have large geographical ranges. There is a somewhat similar phenomena with Cocos-Keeling and Christmas islands, which are grouped with the Central Pacific region in two of our classifications (Figures 4 b, c). This grouping is in partial agreement with Briggs and Bowen [67] who suggest that the Indonesian region is probably a weak barrier with many species crossing. It also helps to explain the presence of hybrids at the boundaries on the Indian Ocean side [71].

None of our four classifications shows the Coral Triangle (or IAA) as a separate entity, which means that there is no strong support in terms of species composition for the delineation of the Coral Triangle (IAA), in agreement with Briggs and Bowen [67] or Kulbicki et al. [63]. The Coral Triangle in our study is either part of a Central Indo-Pacific region (Figures 4 a, b) or makes a province with Melanesia, extending to either the Solomon or Vanuatu archipelagos (Figures 4 c, d). It is, however, important to note that the ‘Coral Triangle’ [8] or IAA [19], [35] is an area distinguished on the basis of species richness not species composition.

Within realms, we found major differences between the Atlantic and ETP on one hand and the Indo-Pacific on the other. In the first two realms, the biogeographical divisions coincide with major barriers, with a clear separation between east and west in the Atlantic and coastal versus offshore islands in the ETP. In the Indo-Pacific there is not the same match between the boundaries of biogeographical regions or provinces and physical barriers. Within the Pacific, the robustness of the branches separating the various regions and provinces is lower than in the other realms (Fig. S1–S4 in File S1) indicating that despite the regions or provinces being distinct, there is a high similarity in species composition amongst them. This high level of similarity is found over most of the Indo-Pacific. For instance, 64% of species are common between Polynesia and the western Indian Ocean despite a distance of over 15,000 km. This huge similarity implies conditions favoring large-scale dispersal and colonization, a pattern that is consistent with evolutionary evidence [36], [62], [63].

Dispersal in the Indian and Pacific Oceans may face different conditions, with the Indian Ocean consisting of large continental masses and a few islands that can be reached via stepping stones, whereas the Pacific is characterized by increasingly smaller and more dispersed islands from west to east. Mora et al. [30] suggested that the distance between these islands is probably not a major obstacle to dispersal for most species. However, the increasingly smaller size of islands results in a smaller and less diverse habitat area [72] and therefore fewer species are able to be supported by these islands [73]. This can be viewed as an attrition process of a species pool as one goes from west to east as already described by Bellwood and Hughes [74]. Therefore, except for local endemics (which are mainly found in the peripheral regions of Hawaii, Easter Island, and the southwestern Pacific), most Indo-Pacific species are from the same initial species pool. This scenario is supported by molecular evidence, with most of the species in the Indian and Pacific Oceans arising as a result of dispersal from lineages within the IAA [36]. As indicated by Bowen et al. [52] this does not exclude the possibility for peripheral regions to export species to the biodiversity hotspots. Indeed the IAA hotspot is both a source of species and location where species from elsewhere can accumulate and survive [36], [52].

Using algorithms based on turnover enhances the delineation of regions with high endemism levels [57]. Our results partially support this pattern with the Indo-Pacific showing a clear separation of provinces with high rates of provincial endemism (>8%) and rather low species numbers (Hawaii −21%, Easter Island- 25%, North-Western Indian Ocean −9.3%, South-West Pacific Ocean −11%). Conversely, regions with high numbers of endemic species associated with high species richness, such as the Indonesian area (199 endemic reef species, 3492 reef species, 5.7% endemism rate), do not systematically constitute specific provinces. This means that our classification does not match with previous works based solely on endemism [4] but does converge for areas of high endemism. It should be noted that our values for endemism are often lower than previously reported [69], in particular for Hawaii [75], Easter Island [76] and the Red Sea [77]. This is probably due to an improved knowledge of species geographical distributions.

The present work is, to our knowledge, the first global quantitative delineation for reef fishes while also accounting for potential sources of uncertainty. The general agreement with previous works on reef fishes at smaller scales leads us to believe that this classification can serve as a useful and robust consensus on the global biogeographical structure for reef fishes. Numerous works have indicated the strong correlation between reef fishes and coral distributions across the globe (see review by Bellwood et al. [25]). Besides corals, a number of studies have highlighted the strong similarities in the patterns of species richness among phyla across the Indo-Pacific [3], [35], [56], [78]. It is therefore probable that the biogeographical regions described herein for reef fishes could be considered as a first proxy for many other organisms.

Our work offers a different perspective when compared to previous global classifications e.g. [5], [8], [11]. For instance, there are many differences with the spatial groups defined by Spalding et al. [11]. These differences are a result of the underlying goals and the methods used. We only used one phylum and the work is based on dissimilarities in species composition, whereas Spalding et al. used many phyla and their delineations were inferred from a combination of expert knowledge, endemism levels and environmental factors. Their work was intended to provide a framework for the management and planning of biodiversity on a global scale, and it may serve many other purposes. Our classification and regional delineations for fishes examined potential weaknesses within our data and can be compared statistically to classifications obtained from other phyla using the same methods. As our knowledge on the distribution of species improves it is likely that some delineations will shift, but looking at the statistical robustness of our results it is unlikely that major shifts will occur, at least within the taxa we examined. The intent of our work is to provide a foundation for developing hypotheses that seek to resolve some of the major questions concerning the evolutionary history and processes underlying fish diversification. Even if there are applications to biodiversity management and planning, this was not the primary scope of our work.

## Conclusion

Our analyses reveal a well-structured hierarchy of biogeographic areas. Three levels were defined, realms, regions, and provinces. The number of clusters was low with only 14 provinces, most of them covering large areas. In particular, the Central Indo-Pacific province, which includes the Coral Triangle or IAA, comprises 58% of the 6,319 reef fish species but covers less than 25% of the area harboring coral reefs. There was a strong contrast between the Atlantic and ETP realms versus the Indo-Pacific realm. The former realms have high levels of endemism and a low diversity at the regional and provincial levels, and therefore low levels of similarity even between closely located provinces. The latter realm displays high diversity, low endemism and extensive faunal overlap resulting in a very high level of similarity from one end of the realm to the other. The choice of a dissimilarity index based on species turnover is important for biogeographical partitioning as the result needs to be independent of species richness, which is an important consideration when analyzing checklists ranging from less than 100 species to over 2,000 species. The analyses and delineations were found to be robust to variation in our level of knowledge of the geographical range of species. The present work is, to our knowledge, the first attempt in the marine world to delineate biogeographical entities based solely on species composition at a global scale. Such a classification complements those based upon endemism, environmental and geographical factors. With a clear delineation of areas we are now in a position to begin to explore the origins and consequences of biogeographical variation in reef fish assemblages.

## Supporting Information

Table S1 **List of the families and genera considered as “reliable”, i.e. for which the geographical distribution is considered as well known.** Only species associated to hard bottoms and reefs were retained.
(DOCX)

File S1 **Supporting Figures S1–S4. Figure S1.** Hierarchical analysis based upon the clustering of species from checklists (this is figure 2 in main text, we reproduce it here so it can be compared with the other dendrograms). All species were kept. This classification is noted as “checklists×all species” in the main text. For clarity the three realms were separated. The values at the start of the branches indicate the proportion of bootstraps (out of 10 000) which yielded the same results. **Figure S2.** Hierarchical analysis based upon the clustering of species from checklists. “Reliable” species were kept. This classification is noted as “checklists×Reliable species” in the main text. For clarity the three realms were separated. The values at the start of the branches indicate the proportion of bootstraps (out of 10 000) which yielded the same results. **Figure S3.** Hierarchical analysis based upon the clustering of species grouped according to the eco-regions defined in Spalding et al. (2007) [11]. All species were kept. This classification is noted as “eco-regions×all species” in the main text. For clarity the three realms were separated. The values at the start of the branches indicate the proportion of bootstraps (out of 10 000) which yielded the same results. **Figure S4.** Hierarchical analysis based upon the clustering of species grouped according to the eco-regions defined in Spalding et al. (2007) [11]. “Reliable” species were kept. This classification is noted as “eco-regions×reliable species” in the main text. For clarity the three realms were separated. The values at the start of the branches indicate the proportion of bootstraps (out of 10 000) which yielded the same results.
(DOCX)
