# Supplementary material for: Global Biogeography of Reef Fishes: A Hierarchical Quantitative Delineation of Regions
Source: PLoS One. 2013 Dec 30;8(12):e81847. doi: 10.1371/journal.pone.0081847 (PMC3875412; doi:10.1371/journal.pone.0081847)
Supplement: Table S1 — List of the families and genera considered as “reliable”, i.e. for which the geographical distribution is considered as well known. Only species associated to hard bottoms and reefs were retained. (DOCX) [file pone.0081847.s001.docx]

**Supplemental Material - I**

List of the families and genera considered as "reliable"

Table S1: List of the families and genera considered as "reliable", i.e. for which the geographical distribution is considered as well known. Only species associated to hard bottoms and reefs were retained.

| Family | Genus | Species | Family | Genus | Species |
| --- | --- | --- | --- | --- | --- |
| Acanthuridae | Acanthurus | 40 | Chaetodontidae | Amphichaetodon | 2 |
| Acanthuridae | Ctenochaetus | 9 | Chaetodontidae | Chaetodon | 87 |
| Acanthuridae | Naso | 19 | Chaetodontidae | Chelmon | 3 |
| Acanthuridae | Paracanthurus | 1 | Chaetodontidae | Chelmonops | 1 |
| Acanthuridae | Prionurus | 7 | Chaetodontidae | Coradion | 3 |
| Acanthuridae | Zebrasoma | 7 | Chaetodontidae | Forcipiger | 2 |
| Aulostomidae | Aulostomus | 3 | Chaetodontidae | Hemitaurichthys | 5 |
| Balistidae | Abalistes | 3 | Chaetodontidae | Heniochus | 8 |
| Balistidae | Balistapus | 1 | Chaetodontidae | Johnrandallia | 1 |
| Balistidae | Balistes | 5 | Chaetodontidae | Parachaetodon | 1 |
| Balistidae | Balistoides | 2 | Chaetodontidae | Prognathodes | 10 |
| Balistidae | Canthidermis | 3 | Chaetodontidae | Roa | 4 |
| Balistidae | Melichthys | 3 | Cheilodactylidae | Cheilodactylus | 5 |
| Balistidae | Odonus | 1 | Cheilodactylidae | Chirodactylus | 3 |
| Balistidae | Pseudobalistes | 3 | Cheilodactylidae | Dactylophora | 1 |
| Balistidae | Rhinecanthus | 6 | Cheilodactylidae | Goniistius | 9 |
| Balistidae | Sufflamen | 5 | Cheilodactylidae | Nemadactylus | 3 |
| Balistidae | Xanthichthys | 6 | Cirrhitidae | Amblycirrhitus | 5 |
| Blenniidae | Meiacanthus | 24 | Cirrhitidae | Cirrhitichthys | 8 |
| Blenniidae | Parablennius | 20 | Cirrhitidae | Cirrhitops | 3 |
| Blenniidae | Plagiotremus | 10 | Cirrhitidae | Cirrhitus | 4 |
| Caesionidae | Caesio | 8 | Cirrhitidae | Cristacirrhitus | 1 |
| Caesionidae | Dipterygonotus | 1 | Cirrhitidae | Cyprinocirrhites | 1 |
| Caesionidae | Gymnocaesio | 1 | Cirrhitidae | Isocirrhitus | 1 |
| Caesionidae | Pterocaesio | 11 | Cirrhitidae | Itycirrhitus | 1 |
| Chaenopsidae | Acanthemblemaria | 18 | Cirrhitidae | Neocirrhites | 1 |
| Chaenopsidae | Chaenopsis | 8 | Cirrhitidae | Notocirrhitus | 1 |
| Chaenopsidae | Cirriemblemaria | 1 | Cirrhitidae | Oxycirrhites | 1 |
| Chaenopsidae | Coralliozetus | 6 | Cirrhitidae | Paracirrhites | 6 |
| Chaenopsidae | Ekemblemaria | 2 | Diodontidae | Allomycterus | 1 |
| Chaenopsidae | Emblemaria | 13 | Diodontidae | Chilomycterus | 7 |
| Chaenopsidae | Emblemariopsis | 10 | Diodontidae | Cyclichthys | 3 |
| Chaenopsidae | Hemiemblemaria | 1 | Diodontidae | Dicotylichthys | 1 |
| Chaenopsidae | Lucayablennius | 1 | Diodontidae | Diodon | 5 |
| Chaenopsidae | Mccoskerichthys | 1 | Diodontidae | Tragulichthys | 1 |
| Chaenopsidae | Neoclinus | 2 | Embiotocidae | Zalembius | 1 |
| Chaenopsidae | Protemblemaria | 3 | Enoplosidae | Enoplosus | 1 |
| Chaenopsidae | Stathmonotus | 6 | Ephippidae | Chaetodipterus | 3 |
| Chaenopsidae | Tanyemblemaria | 1 | Ephippidae | Ephippus | 1 |
| Family | Genus | Species | Family | Genus | Species |
| Ephippidae | Parapsettus | 1 | Labridae | Austrolabrus | 1 |
| Ephippidae | Platax | 5 | Labridae | Bodianus | 37 |
| Ephippidae | Proteracanthus | 1 | Labridae | Centrolabrus | 1 |
| Ephippidae | Tripterodon | 1 | Labridae | Cheilinus | 9 |
| Ephippidae | Zabidius | 1 | Labridae | Cheilio | 1 |
| Gobiidae | Elacatinus | 31 | Labridae | Choerodon | 25 |
| Gobiidae | Valenciennea | 15 | Labridae | Cirrhilabrus | 46 |
| Grammatidae | Gramma | 4 | Labridae | Clepticus | 3 |
| Grammatidae | Lipogramma | 6 | Labridae | Conniella | 1 |
| Haemulidae | Anisotremus | 8 | Labridae | Coris | 26 |
| Haemulidae | Conodon | 1 | Labridae | Cymolutes | 3 |
| Haemulidae | Diagramma | 3 | Labridae | Decodon | 4 |
| Haemulidae | Haemulon | 20 | Labridae | Diproctacanthus | 1 |
| Haemulidae | Microlepidotus | 2 | Labridae | Doratonotus | 1 |
| Haemulidae | Orthopristis | 5 | Labridae | Dotalabrus | 2 |
| Haemulidae | Parapristipoma | 3 | Labridae | Epibulus | 2 |
| Haemulidae | Plectorhinchus | 27 | Labridae | Eupetrichthys | 1 |
| Haemulidae | Pomadasys | 21 | Labridae | Gomphosus | 2 |
| Haemulidae | Xenichthys | 3 | Labridae | Haletta | 1 |
| Haemulidae | Xenistius | 1 | Labridae | Halichoeres | 76 |
| Haemulidae | Xenocys | 1 | Labridae | Hemigymnus | 2 |
| Holocentridae | Sargocentron | 32 | Labridae | Hologymnosus | 4 |
| Kyphosidae | Atypichthys | 1 | Labridae | Iniistius | 8 |
| Kyphosidae | Bathystethus | 2 | Labridae | Labrichthys | 1 |
| Kyphosidae | Girella | 15 | Labridae | Labroides | 5 |
| Kyphosidae | Hermosilla | 1 | Labridae | Labropsis | 6 |
| Kyphosidae | Kyphosus | 13 | Labridae | Labrus | 2 |
| Kyphosidae | Labracoglossa | 2 | Labridae | Lachnolaimus | 1 |
| Kyphosidae | Medialuna | 1 | Labridae | Lappanella | 1 |
| Kyphosidae | Microcanthus | 1 | Labridae | Larabicus | 1 |
| Kyphosidae | Neatypus | 1 | Labridae | Leptojulis | 4 |
| Kyphosidae | Neoscorpis | 1 | Labridae | Macropharyngodon | 10 |
| Kyphosidae | Scorpis | 4 | Labridae | Minilabrus | 1 |
| Kyphosidae | Sectator | 1 | Labridae | Notolabrus | 5 |
| Labridae | Acantholabrus | 1 | Labridae | Novaculichthys | 2 |
| Labridae | Achoerodus | 1 | Labridae | Odax | 2 |
| Labridae | Ammolabrus | 1 | Labridae | Ophthalmolepis | 1 |
| Labridae | Anampses | 13 | Labridae | Oxycheilinus | 8 |
| Labridae | Anchichoerops | 1 | Labridae | Paracheilinus | 16 |
| Labridae | Parajulis | 1 | Lutjanidae | Symphorichthys | 1 |
| Labridae | Pictilabrus | 2 | Lutjanidae | Symphorus | 1 |
| Labridae | Polylepion | 2 | Malacanthidae | Branchiostegus | 10 |
| Labridae | Pseudocheilinops | 1 | Malacanthidae | Caulolatilus | 3 |
| Labridae | Pseudocheilinus | 7 | Malacanthidae | Hoplolatilus | 10 |
| Labridae | Pseudocoris | 5 | Malacanthidae | Lopholatilus | 1 |
| Labridae | Pseudodax | 1 | Malacanthidae | Malacanthus | 3 |
| Labridae | Pseudojuloides | 10 | Microdesmidae | Cerdale | 3 |
| Family | Genus | Species | Family | Genus | Species |
| Labridae | Pseudolabrus | 10 | Microdesmidae | Clarkichthys | 1 |
| Labridae | Pteragogus | 7 | Microdesmidae | Gunnellichthys | 6 |
| Labridae | Semicossyphus | 3 | Microdesmidae | Microdesmus | 5 |
| Labridae | Siphonognathus | 5 | Microdesmidae | Paragunnellichthys | 3 |
| Labridae | Stethojulis | 10 | Monacanthidae | Acanthaluteres | 3 |
| Labridae | Suezichthys | 8 | Monacanthidae | Acreichthys | 2 |
| Labridae | Symphodus | 2 | Monacanthidae | Aluterus | 4 |
| Labridae | Terelabrus | 1 | Monacanthidae | Amanses | 1 |
| Labridae | Thalassoma | 28 | Monacanthidae | Anacanthus | 1 |
| Labridae | Wetmorella | 3 | Monacanthidae | Brachaluteres | 4 |
| Labridae | Xenojulis | 1 | Monacanthidae | Cantherhines | 12 |
| Labridae | Xiphocheilus | 1 | Monacanthidae | Cantheschenia | 2 |
| Labridae | Xyrichtys | 22 | Monacanthidae | Chaetodermis | 1 |
| Labrisomidae | Labrisomus | 18 | Monacanthidae | Eubalichthys | 4 |
| Lethrinidae | Gnathodentex | 1 | Monacanthidae | Meuschenia | 6 |
| Lethrinidae | Gymnocranius | 7 | Monacanthidae | Monacanthus | 3 |
| Lethrinidae | Lethrinus | 27 | Monacanthidae | Nelusetta | 1 |
| Lethrinidae | Monotaxis | 2 | Monacanthidae | Oxymonacanthus | 2 |
| Lutjanidae | Aphareus | 2 | Monacanthidae | Paraluteres | 2 |
| Lutjanidae | Aprion | 1 | Monacanthidae | Paramonacanthus | 5 |
| Lutjanidae | Apsilus | 2 | Monacanthidae | Pervagor | 8 |
| Lutjanidae | Etelis | 1 | Monacanthidae | Pseudalutarius | 1 |
| Lutjanidae | Hoplopagrus | 1 | Monacanthidae | Pseudomonacanthus | 3 |
| Lutjanidae | Lipocheilus | 1 | Monacanthidae | Rudarius | 2 |
| Lutjanidae | Lutjanus | 62 | Monacanthidae | Scobinichthys | 1 |
| Lutjanidae | Macolor | 2 | Monacanthidae | Stephanolepis | 5 |
| Lutjanidae | Ocyurus | 1 | Monacanthidae | Thamnaconus | 5 |
| Lutjanidae | Paracaesio | 2 | Monocentridae | Cleidopus | 1 |
| Lutjanidae | Pinjalo | 2 | Monocentridae | Monocentris | 1 |
| Lutjanidae | Pristipomoides | 3 | Mullidae | Mulloidichthys | 6 |
| Lutjanidae | Rhomboplites | 1 | Mullidae | Mullus | 4 |
| Mullidae | Parupeneus | 29 | Pomacentridae | Chromis | 87 |
| Mullidae | Pseudupeneus | 3 | Pomacentridae | Chrysiptera | 34 |
| Mullidae | Upeneichthys | 3 | Pomacentridae | Dascyllus | 10 |
| Mullidae | Upeneus | 7 | Pomacentridae | Dischistodus | 7 |
| Nemipteridae | Nemipterus | 1 | Pomacentridae | Hemiglyphidodon | 1 |
| Nemipteridae | Pentapodus | 10 | Pomacentridae | Hypsypops | 1 |
| Nemipteridae | Scaevius | 1 | Pomacentridae | Lepidozygus | 1 |
| Nemipteridae | Scolopsis | 14 | Pomacentridae | Microspathodon | 4 |
| Oplegnathidae | Oplegnathus | 6 | Pomacentridae | Neoglyphidodon | 8 |
| Ostraciidae | Acanthostracion | 4 | Pomacentridae | Neopomacentrus | 14 |
| Ostraciidae | Anoplocapros | 3 | Pomacentridae | Nexilosus | 1 |
| Ostraciidae | Aracana | 2 | Pomacentridae | Parma | 9 |
| Ostraciidae | Kentrocapros | 1 | Pomacentridae | Plectroglyphidodon | 10 |
| Ostraciidae | Lactophrys | 3 | Pomacentridae | Pomacentrus | 71 |
| Ostraciidae | Lactoria | 3 | Pomacentridae | Pomachromis | 3 |
| Ostraciidae | Ostracion | 9 | Pomacentridae | Premnas | 1 |
| Family | Genus | Species | Family | Genus | Species |
| Ostraciidae | Tetrosomus | 2 | Pomacentridae | Pristotis | 2 |
| Pentacerotidae | Evistias | 1 | Pomacentridae | Similiparma | 1 |
| Pentacerotidae | Histiopterus | 1 | Pomacentridae | Stegastes | 37 |
| Pentacerotidae | Pentaceros | 1 | Pomacentridae | Teixeirichthys | 1 |
| Pinguipedidae | Parapercis | 45 | Scaridae | Bolbometopon | 1 |
| Pinguipedidae | Pinguipes | 1 | Scaridae | Calotomus | 5 |
| Pinguipedidae | Pseudopercis | 2 | Scaridae | Cetoscarus | 2 |
| Pinguipedidae | Ryukyupercis | 1 | Scaridae | Chlorurus | 17 |
| Pomacanthidae | Apolemichthys | 8 | Scaridae | Cryptotomus | 1 |
| Pomacanthidae | Centropyge | 33 | Scaridae | Hipposcarus | 2 |
| Pomacanthidae | Chaetodontoplus | 14 | Scaridae | Leptoscarus | 1 |
| Pomacanthidae | Genicanthus | 10 | Scaridae | Nicholsina | 3 |
| Pomacanthidae | Holacanthus | 7 | Scaridae | Scarus | 49 |
| Pomacanthidae | Pomacanthus | 13 | Scaridae | Sparisoma | 13 |
| Pomacanthidae | Pygoplites | 1 | Sciaenidae | Argyrosomus | 3 |
| Pomacentridae | Abudefduf | 20 | Sciaenidae | Atractoscion | 1 |
| Pomacentridae | Acanthochromis | 1 | Sciaenidae | Bairdiella | 1 |
| Pomacentridae | Altrichthys | 2 | Sciaenidae | Cheilotrema | 1 |
| Pomacentridae | Amblyglyphidodon | 10 | Sciaenidae | Corvula | 1 |
| Pomacentridae | Amblypomacentrus | 2 | Sciaenidae | Equetus | 2 |
| Pomacentridae | Amphiprion | 30 | Sciaenidae | Odontoscion | 3 |
| Pomacentridae | Azurina | 2 | Sciaenidae | Otolithes | 1 |
| Pomacentridae | Cheiloprion | 1 | Sciaenidae | Pareques | 6 |
| Sciaenidae | Pseudotolithus | 1 | Serranidae | Othos | 1 |
| Sciaenidae | Totoaba | 1 | Serranidae | Paralabrax | 8 |
| Sciaenidae | Umbrina | 5 | Serranidae | Paranthias | 2 |
| Sebastidae | Sebastes | 1 | Serranidae | Parasphyraenops | 1 |
| Serranidae | Acanthistius | 9 | Serranidae | Plectranthias | 18 |
| Serranidae | Aethaloperca | 1 | Serranidae | Plectropomus | 7 |
| Serranidae | Alphestes | 3 | Serranidae | Pogonoperca | 2 |
| Serranidae | Anthias | 5 | Serranidae | Pronotogrammus | 3 |
| Serranidae | Anyperodon | 1 | Serranidae | Pseudanthias | 56 |
| Serranidae | Aporops | 1 | Serranidae | Pseudogramma | 11 |
| Serranidae | Aulacocephalus | 1 | Serranidae | Rabaulichthys | 2 |
| Serranidae | Belonoperca | 1 | Serranidae | Rainfordia | 1 |
| Serranidae | Caesioscorpis | 1 | Serranidae | Rypticus | 9 |
| Serranidae | Caprodon | 3 | Serranidae | Sacura | 2 |
| Serranidae | Centropristis | 4 | Serranidae | Schultzea | 1 |
| Serranidae | Cephalopholis | 22 | Serranidae | Serraniculus | 1 |
| Serranidae | Cromileptes | 1 | Serranidae | Serranocirrhitus | 1 |
| Serranidae | Dermatolepis | 3 | Serranidae | Serranus | 27 |
| Serranidae | Diplectrum | 3 | Serranidae | Suttonia | 2 |
| Serranidae | Diploprion | 2 | Serranidae | Tosanoides | 1 |
| Serranidae | Epinephelides | 1 | Serranidae | Trachypoma | 1 |
| Serranidae | Epinephelus | 84 | Serranidae | Triso | 1 |
| Serranidae | Giganthias | 1 | Serranidae | Variola | 2 |
| Serranidae | Gonioplectrus | 1 | Siganidae | Siganus | 28 |
| Family | Genus | Species | Family | Genus | Species |
| Serranidae | Gracila | 1 | Sparidae | Acanthopagrus | 5 |
| Serranidae | Grammistes | 1 | Sparidae | Archosargus | 3 |
| Serranidae | Grammistops | 1 | Sparidae | Argyrops | 3 |
| Serranidae | Hemanthias | 2 | Sparidae | Argyrozona | 1 |
| Serranidae | Hemilutjanus | 1 | Sparidae | Boops | 2 |
| Serranidae | Holanthias | 2 | Sparidae | Boopsoidea | 1 |
| Serranidae | Hypoplectrodes | 6 | Sparidae | Calamus | 12 |
| Serranidae | Hypoplectrus | 10 | Sparidae | Cheimerius | 1 |
| Serranidae | Lepidoperca | 1 | Sparidae | Chrysoblephus | 6 |
| Serranidae | Liopropoma | 23 | Sparidae | Cymatoceps | 1 |
| Serranidae | Luzonichthys | 6 | Sparidae | Dentex | 7 |
| Serranidae | Mycteroperca | 15 | Sparidae | Diplodus | 19 |
| Serranidae | Nemanthias | 1 | Sparidae | Evynnis | 2 |
| Serranidae | Niphon | 1 | Sparidae | Gymnocrotaphus | 1 |
| Serranidae | Odontanthias | 2 | Sparidae | Lagodon | 1 |
| Sparidae | Lithognathus | 1 | Sparidae | Virididentex | 1 |
| Sparidae | Oblada | 1 | Tetraodontidae | Amblyrhynchotes | 1 |
| Sparidae | Pachymetopon | 3 | Tetraodontidae | Arothron | 13 |
| Sparidae | Pagellus | 6 | Tetraodontidae | Canthigaster | 31 |
| Sparidae | Pagrus | 6 | Tetraodontidae | Chelonodon | 4 |
| Sparidae | Polyamblyodon | 2 | Tetraodontidae | Ephippion | 1 |
| Sparidae | Polysteganus | 2 | Tetraodontidae | Feroxodon | 1 |
| Sparidae | Porcostoma | 1 | Tetraodontidae | Guentheridia | 1 |
| Sparidae | Pterogymnus | 1 | Tetraodontidae | Javichthys | 1 |
| Sparidae | Rhabdosargus | 3 | Tetraodontidae | Lagocephalus | 3 |
| Sparidae | Sarpa | 1 | Tetraodontidae | Omegophora | 2 |
| Sparidae | Sparidentex | 1 | Tetraodontidae | Pelagocephalus | 1 |
| Sparidae | Sparodon | 1 | Tetraodontidae | Sphoeroides | 9 |
| Sparidae | Sparus | 1 | Tetraodontidae | Takifugu | 3 |
| Sparidae | Spondyliosoma | 2 | Tetraodontidae | Torquigener | 2 |
| Sparidae | Stenotomus | 2 | Zanclidae | Zanclus | 1 |
