# Supplementary material for: Global Biogeography of Reef Fishes: A Hierarchical Quantitative Delineation of Regions
Source: PLoS One. 2013 Dec 30;8(12):e81847. doi: 10.1371/journal.pone.0081847 (PMC3875412; doi:10.1371/journal.pone.0081847)
Supplement: File S1 — Supporting Figures S1–S4. Figure S1. Hierarchical analysis based upon the clustering of species from checklists (this is figure 2 in main text, we reproduce it here so it can be compared with the other dendrograms). All species were kept. This classification is noted as “checklists×all species” in the main text. For clarity the three realms were separated. The values at the start of the branches indicate the proportion of bootstraps (out of 10 000) which yielded the same results. Figure S2. Hierarchical analysis based upon the clustering of species from checklists. “Reliable” species were kept. This classification is noted as “checklists×Reliable species” in the main text. For clarity the three realms were separated. The values at the start of the branches indicate the proportion of bootstraps (out of 10 000) which yielded the same results. Figure S3. Hierarchical analysis based upon the clustering of species grouped according to the eco-regions defined in Spalding et al. (2007) [11]. All species were kept. This classification is noted as “eco-regions×all species” in the main text. For clarity the three realms were separated. The values at the start of the branches indicate the proportion of bootstraps (out of 10 000) which yielded the same results. Figure S4. Hierarchical analysis based upon the clustering of species grouped according to the eco-regions defined in Spalding et al. (2007) [11]. “Reliable” species were kept. This classification is noted as “eco-regions×reliable species” in the main text. For clarity the three realms were separated. The values at the start of the branches indicate the proportion of bootstraps (out of 10 000) which yielded the same results. (DOCX) [file pone.0081847.s002.docx]

**Supplemental Material II**

Clusters from the four analyses: 1- all species X checklists; 2: reliable species X checklists; 3: all species X eco-regions; 4- reliable species X eco-regions


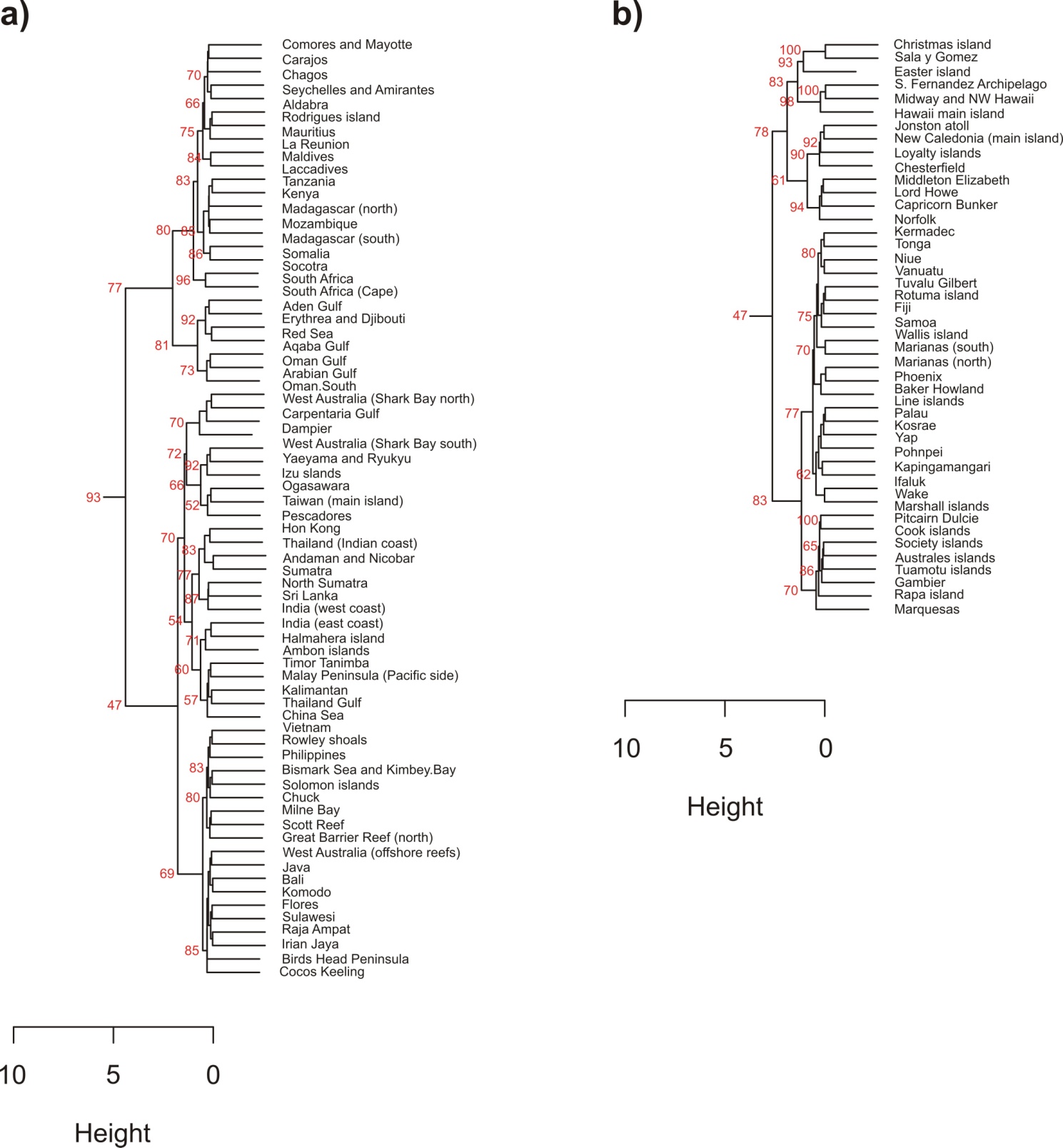


**Central Pacific**

**Central Indo-Pacific**

**Western Indian Ocean**


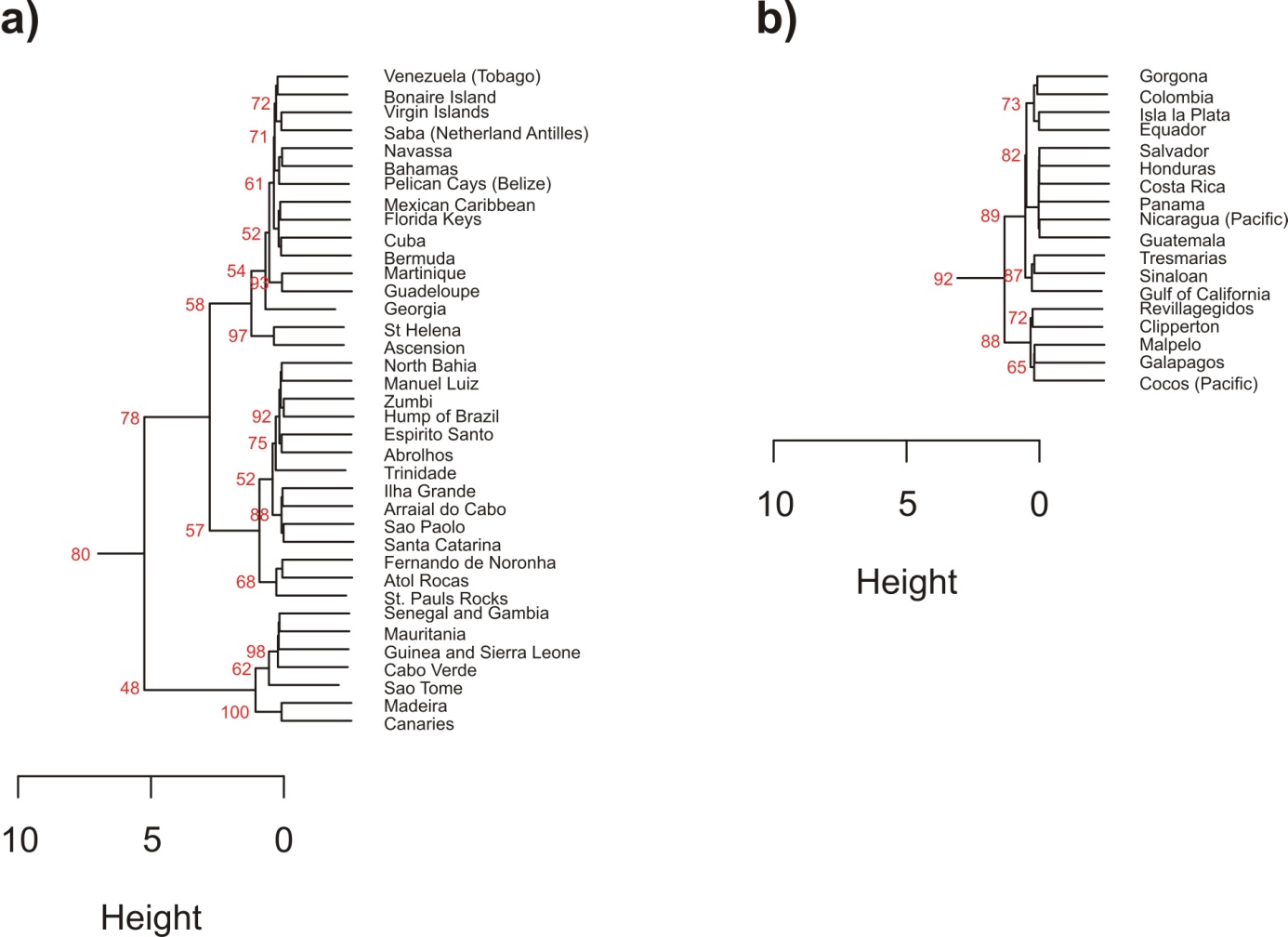


**d)**

**c)**

**Eastern Tropical Pacific**

**Atlantic**

Fig S1- Hierarchical analysis based upon the clustering of species from checklists (this is figure 2 in main text, we reproduce it here so it can be compared with the other dendrograms). All species were kept. This classification is noted as "checklists X all species" in the main text. For clarity the three realms were separated. The values at the start of the branches indicate the proportion of bootstraps (out of 10 000) which yielded the same results.


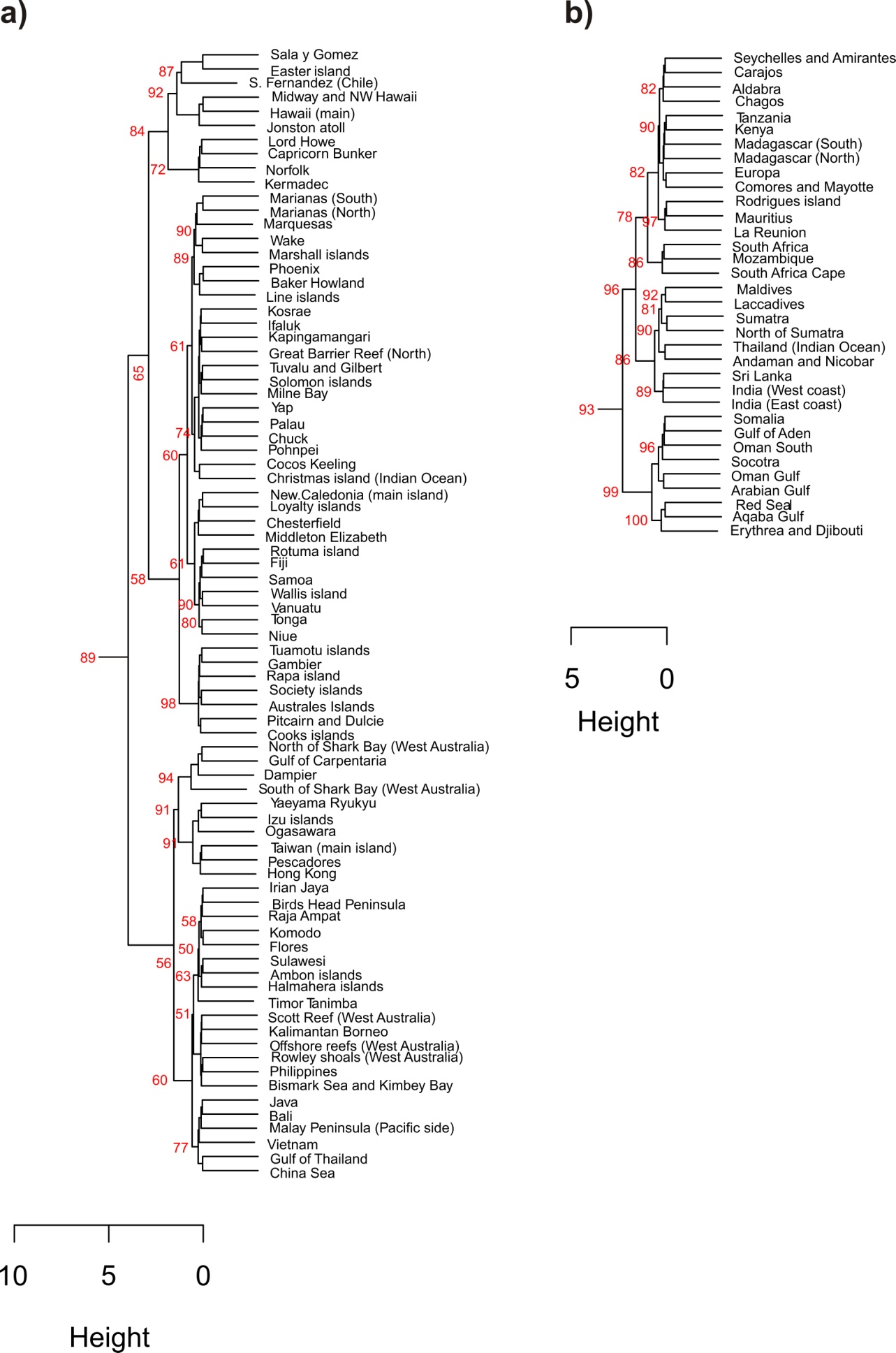


**Central Pacific**

**Central Indo-Pacific**

**Western Indian Ocean**


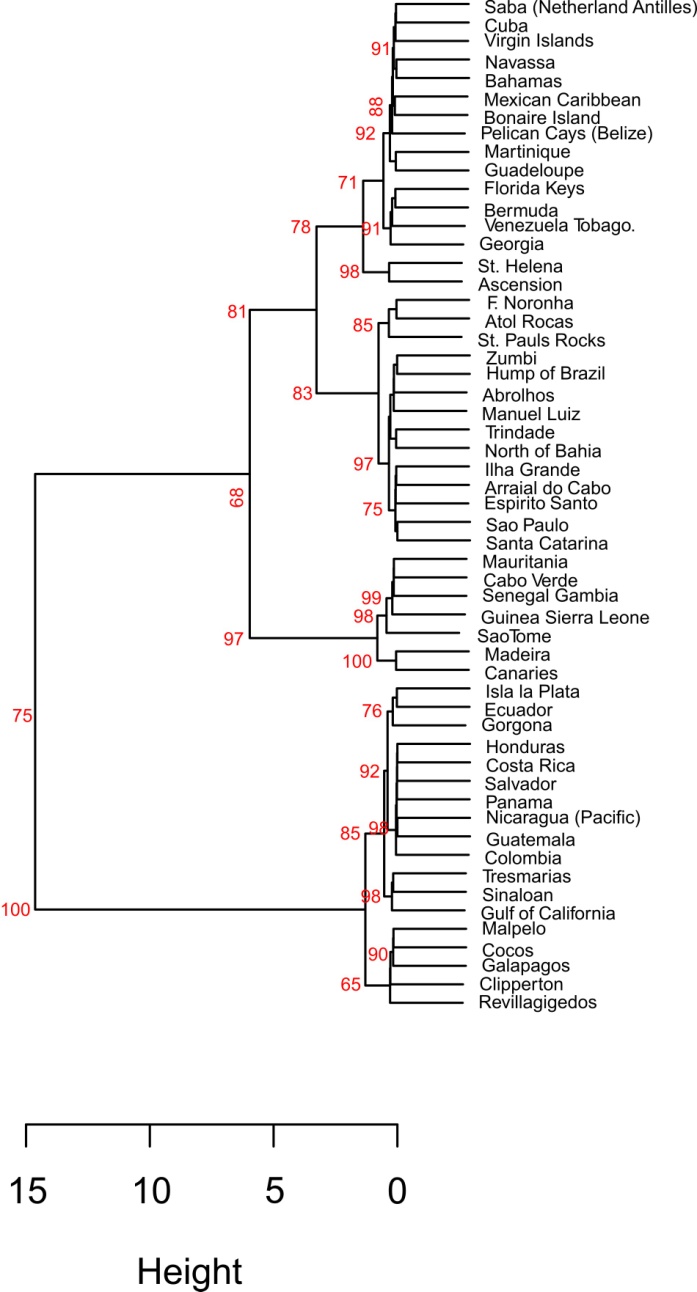


**Eastern Tropical Pacific**

**Atlantic**

Fig. S2- Hierarchical analysis based upon the clustering of species from checklists. "Reliable" species were kept. This classification is noted as "checklists X Reliable species" in the main text. For clarity the three realms were separated. The values at the start of the branches indicate the proportion of bootstraps (out of 10 000) which yielded the same results.

**Western Indian Ocean**


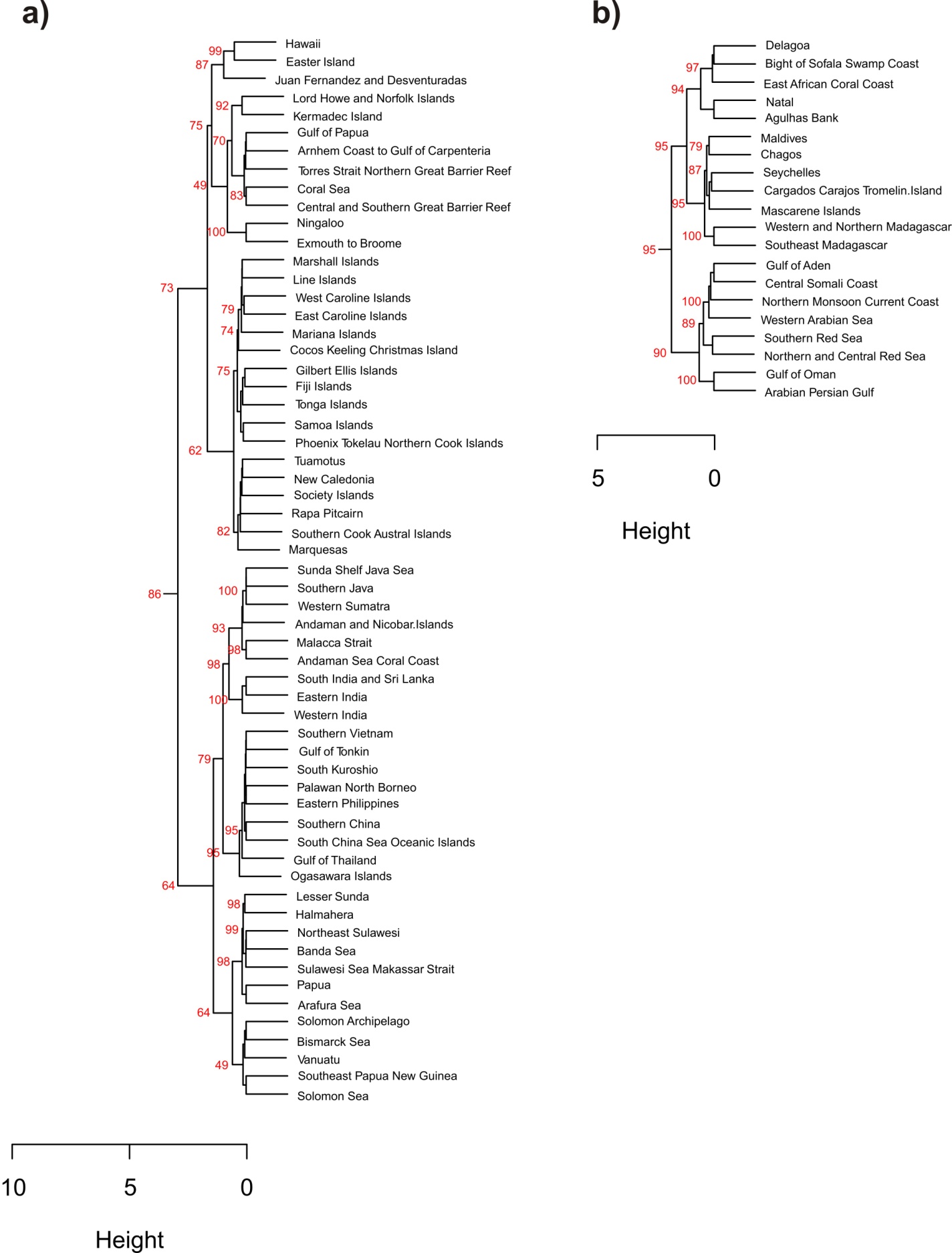


**Pacific & East Indian Oceans**

**Ocean**


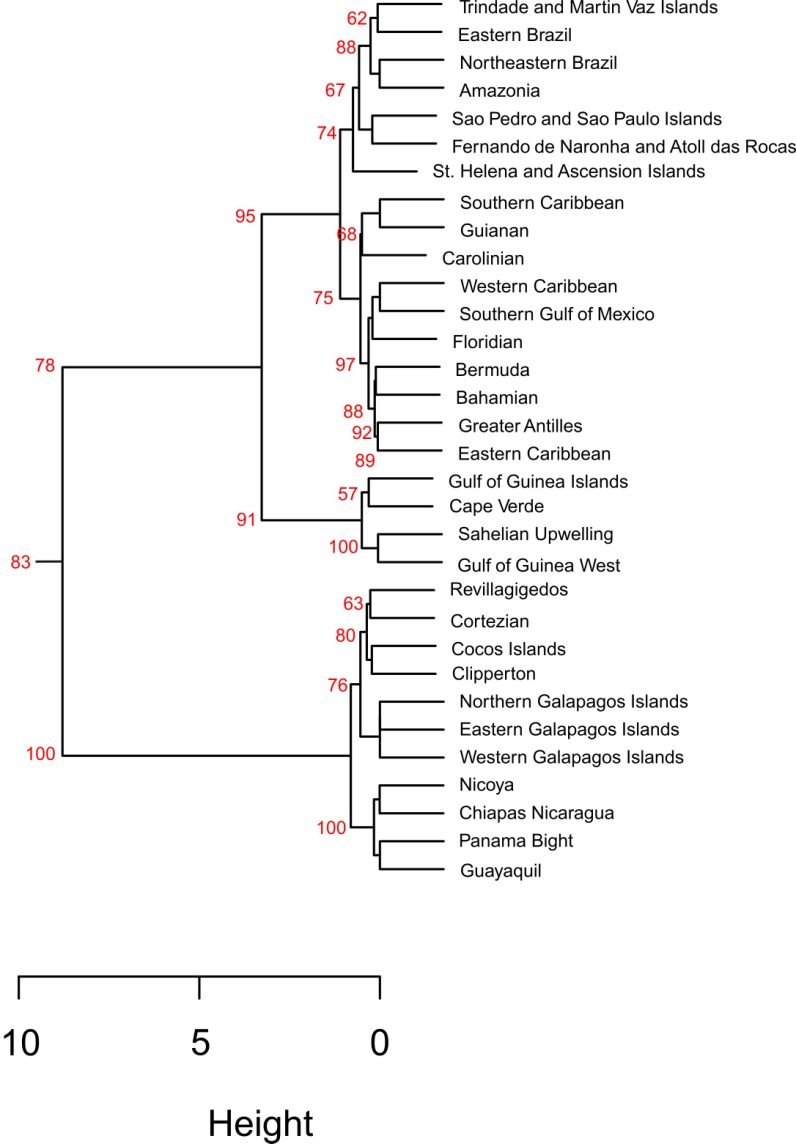


**Eastern Tropical Pacific**

**Atlantic**

Fig S3- Hierarchical analysis based upon the clustering of species grouped according to the eco-regions defined in Spalding et al. (2007). All species were kept. This classification is noted as "eco-regions X all species" in the main text. For clarity the three realms were separated. The values at the start of the branches indicate the proportion of bootstraps (out of 10 000) which yielded the same results.


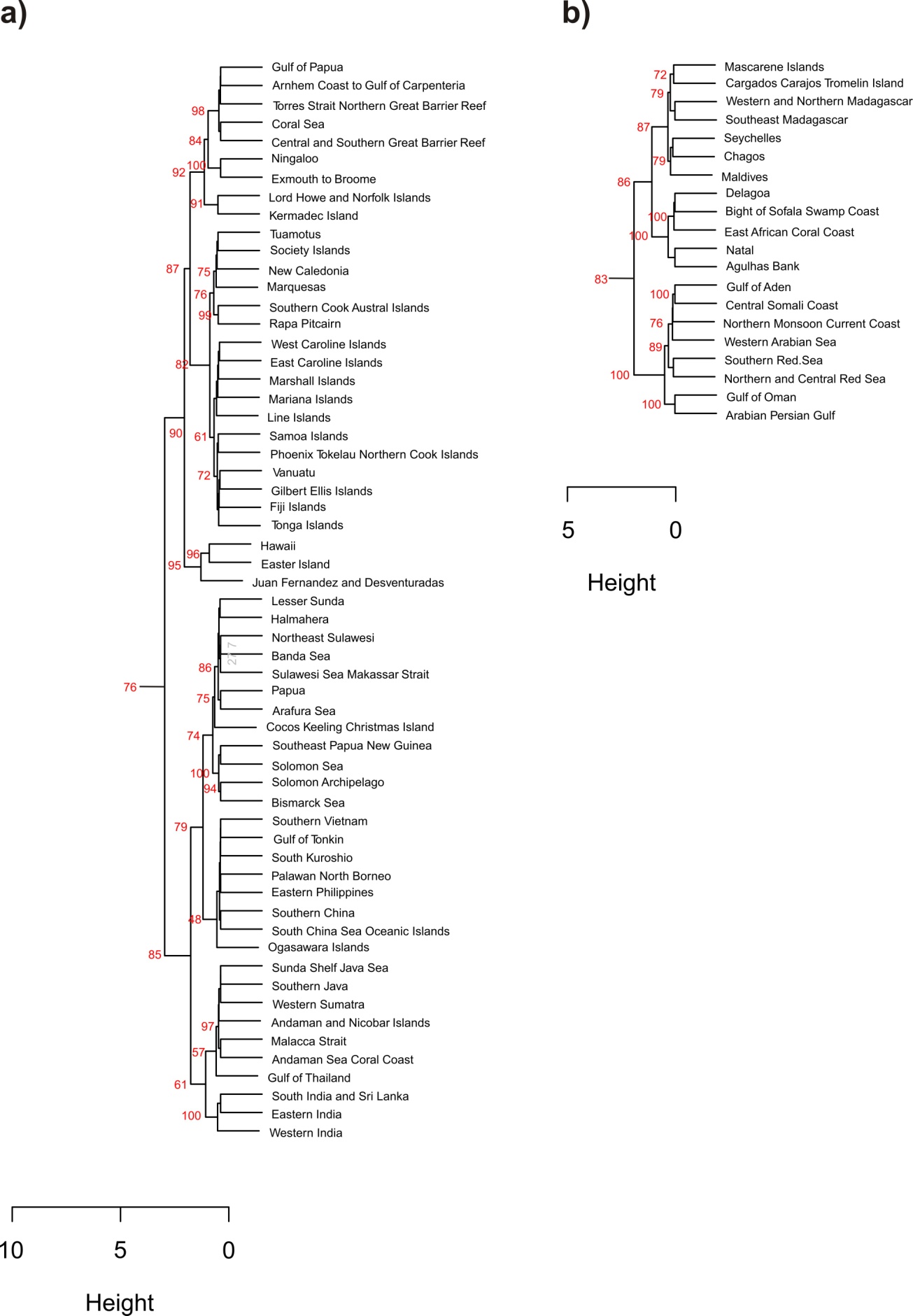


**Pacific & East Indian Oceans**

**Ocean**

**Western Indian Ocean**


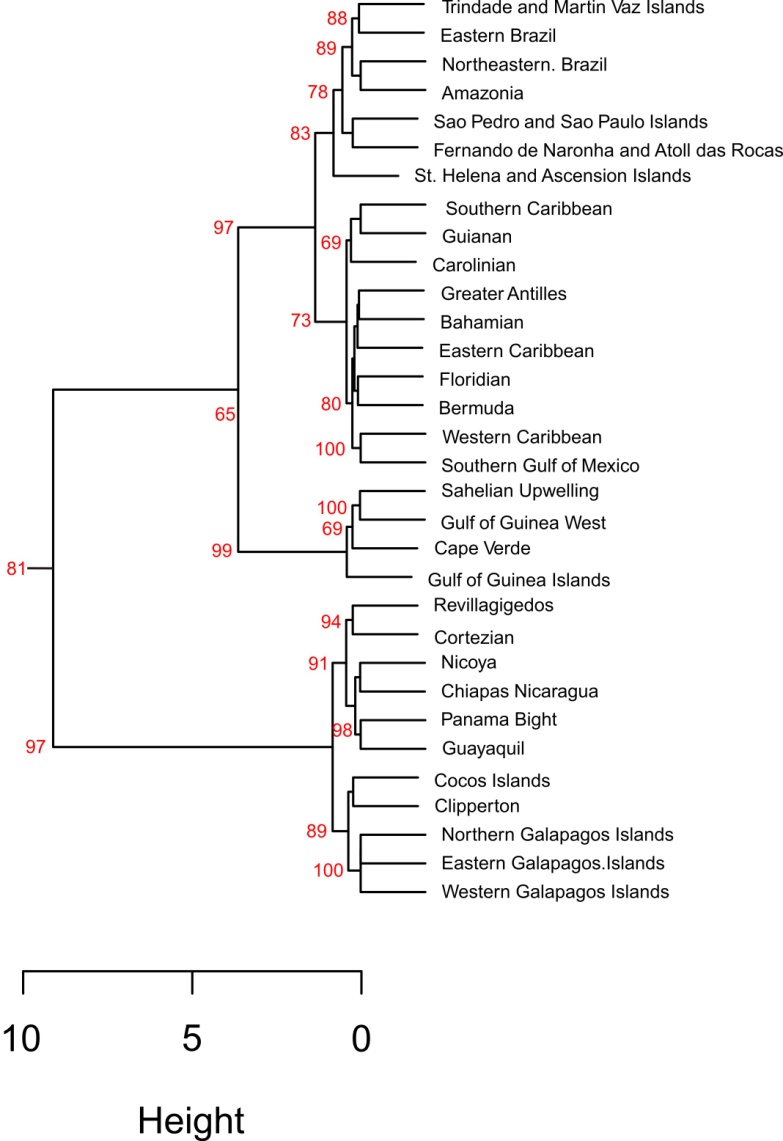


**Eastern Tropical Pacific**

**Atlantic**

Fig. S4- Hierarchical analysis based upon the clustering of species grouped according to the eco-regions defined in Spalding et al. (2007). "Reliable" species were kept. This classification is noted as "eco-regions X reliable species" in the main text. For clarity the three realms were separated. The values at the start of the branches indicate the proportion of bootstraps (out of 10 000) which yielded the same results.
